# Protective Effects of Live Combined *B. subtilis* and *E. faecium* in Polymicrobial Sepsis Through Modulating Activation and Transformation of Macrophages and Mast Cells

**DOI:** 10.3389/fphar.2018.01506

**Published:** 2019-01-21

**Authors:** Lisha Guo, Mei Meng, Yaping Wei, Feixue Lin, Ying Jiang, Xianzhen Cui, Guirong Wang, Chunting Wang, Xiaosun Guo

**Affiliations:** ^1^Department of Critical Care Medicine, Shandong Provincial Hospital Affiliated to Shandong University, Jinan, China; ^2^Department of Emergency, Binzhou Medical University Hospital, Binzhou, China; ^3^Department of Physiology and Pathophysiology, School of Basic Medicine, Shandong University, Jinan, China; ^4^Binzhou Medical University Hospital, Binzhou, China; ^5^School of Medicine, Shandong University, Jinan, China; ^6^Department of Surgery, SUNY Upstate Medical University, Syracuse, NY, United States

**Keywords:** AKT pathway, live combined *Bacillus subtilis* and *Enterococcus faecium* (LCBE) enteric-coated capsules, macrophage activation and transformation, mast cell degranulation, CLP sepsis

## Abstract

**Aims:** Clinical studies showed that the use of probiotics during critical illness reduced nosocomial infection and improved clinical outcome. However, the functional mechanisms of probiotics is remains unclear. Therefore the aim of current study is to explore the protective effects and understand the underlying mechanisms for the beneficial effects of live combined *Bacillus subtilis* and *Enterococcus faecium* (LCBE) in cecal ligation puncture (CLP)-induced sepsis.

**Methods and Results:** Seven-week-old C57BL/6J mice were divided into three groups: sham group (6 mice), CLP-control group (20 mice, pretreatment with saline for 7 days before CLP surgery) and CLP-probiotics group (14 mice, pretreatment with LCBE enteric-coated capsules for 7 days before CLP surgery). In survival experiment, mice were monitored for 7 days after CLP. After the procedure, mice were sacrificed, and, serum, and peritoneal lavage fluid were collected and intestinal ileal samples were harvested.

**Results:** Our results showed that the mortality was significantly reduced in mice CLP-probiotics group vs. CLP-control group (*P* < 0.05). Also, treatment CLP-probiotics group decreased the injury scores CLP-probiotics group when compared to CLP-control group. Additionally, levels of pro-inflammatory cytokines IL-6 and TNF-α levels in the serum and intestinal ileal tissues of CLP-probiotics group were reduced when compared to CLP-control group (*P* < 0.05). However, no significant differences in anti-inflammatory levels of IL-10 and TGF-β1 were observed between CLP-control and CLP-probiotic groups. Furthermore, our experiments showed that that probiotic treatment suppressed the macrophage activation and transformation from M-type to M1-type, inhibited the mast cells (MCs) degranulation, and activation of AKT (kinase B) pathway.

**Conclusion:** In conclusion, our data shows that probiotics have a protective role in CLP septic mice through reducing intestinal inflammation, altering macrophage polarization and MCs degranulation, and regulating AKT signaling.

**Significance and Impact of Study:** This study demonstrated the protective effects and mechanisms involved in the protective role of live combined *Bacillus subtilis* and *Enterococcus faecium* (LCBE) in CLP-induced septic mice model.

## Introduction

Sepsis is life threatening organ dysfunction caused by a dysregulated host response to infection, and continues to be the leading cause of mortality in the intensive care unit in developed countries (Hotchkiss et al., 2013; Vincent et al., 2013; Deutschman et al., 2016). Accumulating evidence showed that abnormal host immune responses, inflammatory cytokines trigger of a “cytokine storm” resulting in subsequent systemic inflammatory response syndrome (SIRS), septic shock, and multiple organ dysfunction syndrome (MODS) and death (Höflich and Volk, 2008; Hotchkiss et al., 2013). Significant advances have been made in understanding the pathogenesis of sepsis, development of new therapeutic agent toll-like receptor 4 antagonists that have been evaluated in clinical trials, however, there are only few successful results (Fink and Warren, 2014; Kuzmich et al., 2017). Patients with severe sepsis usually have severe injury in their gastrointestinal system (Mittal and Coopersmith, 2014; Klingensmith and Coopersmith, 2016). Therefore, it is essential to study the underlying mechanisms of sepsis-induced gastrointestinal injury and develop novel therapeutic strategies to decrease the morbidity and mortality in septic patients.

The gastrointestinal tract has long been hypothesized to play an integral role in the pathophysiology of sepsis, by acting as a motor that both drives and perpetuates multiple organ dysfunction. The gastrointestinal tract, a highly specialized intrinsic immune system, possesses the highest concentration of immune cells in the human body to maintain homeostasis and protect the body from incoming pathogens (Clark and Coopersmith, 2007). In the past decades, numerous studies have reported that macrophages and mast cells (MCs) were implicated in the mediation of sepsis by the modulation of inflammatory and immune responses in a mouse cecal ligation puncture (CLP) model (Gautier et al., 2014; Gautier and Launay, 2015). For example, previous studies demonstrated that macrophages increased acute lung injury (ALI) through increased expression of macrophage inhibitory factor (MIF) in a sepsis-induced ALI rat model (Wang et al., 2014). MCs increases the recruitment of neutrophils through release of several inflammatory mediators that includes tumor necrosis factor (TNF), histamine and leukotrienes, and reduced animal survival in lipopolysaccharide (LPS)-induced sepsis rodent model (Liboni et al., 2005). However, the exact role of macrophages, remain unclear in sepsis.

The human intestinal microbiota, composed of 10^13^ to 10^14^ microorganisms that play an important role in epithelial barrier and gut immune system (Dou and Bennett, 2017). Among the intestinal microbiota, probiotics that includes *Bacillus subtilis*, Bifidobacterium, Lactobacillus, and Enterococcus are living microorganisms that have beneficial effects on the host (Lievin-Le Moal and Servin, 2014). A growing body of experimental and clinical evidence has shown that probiotics exert their protective roles through inhibition of pathogen adhesion to intestinal surface, thereby improving function of gut epithelial barrier, and modulates immune function through regulation of secretion of various inflammatory mediators in several inflammatory bowel, infectious, gastrointestinal diseases (Markowiak and Slizewska, 2017). Therefore, probiotics could be used as a potential agent for the treatment of sepsis-induced gastrointestinal diseases, due to the barrier integrity and permeability of intestine. The expression of the tight junction (TJ) proteins decreased during weaning, and as a result, barrier integrity was impaired. This facilitated pathogen penetration and permits bacteriotoxins to enter the body. The consumption of probiotic bacteria contributes to intestinal function by maintaining paracellular permeability, enhancing the physical mucous layer, stimulating the immune system, and modulating resident microbiota composition and activity (Boirivant and Strober, 2007). Recently, Chen et al. (2016) have reported that probiotic pre-administration could significantly reduce the mortality rate in a mouse model of CLP-induced sepsis. However, the underlying mechanisms of probiotics in regulating sepsis-induced gastrointestinal injury have not been elucidated. In this study, we examined the protective effects of probiotics in CLP-induced sepsis, and also studied their role in on macrophage activation and transformation, MC degranulation, and AKT signaling activation.

## Materials and Methods

### CLP-Induced Sepsis and Treatment With Probiotics

Seven-week-old male C57BL/6J mice, approximately 22 g body weight, were purchased from Beijing Vito Lihua Company, and housed in the animal facility of Shandong University for acclimatization for at least 1 week before use. All mice were provided with free access to autoclaved food and water under specific pathogen-free conditions with an alternating 12 h light/dark cycle at 25 ± 2°C. All experimental procedures were compliant with the Guidelines for Laboratory Animal Use and Care from the Chinese Center for Disease Control and Prevention and the Rules for Medical Laboratory Animals from the Chinese Ministry of Health, under protocol CAU-AEC-2013-073 approved by the Animal Ethics and Care Committee of Shandong University. All experiments were carried out in accordance with the National Institutes of Health and ARRIVE guidelines on the use of laboratory animals.

After 6 h of chow and water were removed, mice were orally gavaged with 200 μl/day probiotic capsule (400 mg/kg), containing 250 mg inulin powder and 0.5 × 10^8^ colony forming unit (CFU) of *B. subtilis* and 4.5 × 10^8^ CFU of *E. faecium* (Beijing Hanmi Pharmaceutical Co., Ltd., China), or normal saline 1 week prior to perform CLP surgery. Subsequently, mice were randomly divided into three groups: Sham group, CLP-control group and CLP-probiotics group, and CLP surgery was performed as described below. Briefly, mice were firstly anesthetized with 1% phenobarbital sodium (40 mg/kg) by intraperitoneal injection and then a 1.0 cm median laparotomy incision was opened, the cecum was isolated, and the distal part of ileocecal valve/cecum was ligated with a 4-0 silk thread, without disrupting bowel continuity. The ligated cecum was punctured twice with an 18-gauge needle, and a small amount of feces was extruded to ensure the patency of the puncture site before returning it back to the abdominal cavity. Sham-operated animals underwent an identical surgical procedure including laparotomy and mobilization of the cecum, but without CLP. The mice were provide with phosphate-buffered saline (PBS, 50 ml/kg body weight) were subcutaneously injected. Postoperative pain was managed with intraperitoneal injection of meloxicam (1.5 mg/kg; R&D systems, United States), ceftriaxone (50 mg/kg; R&D systems, United States) and Meronidal (50 mg/kg; R&D systems, United States) every 12 h for 2 days (Echtenacher et al., 1996; Mallen–St Clair et al., 2004).

### Assessment of Mortality Rate

Survival studies were conducted to determine whether probiotic capsule [live combined *B. subtilis* and *E. faecium* (LCBE) enteric-coated capsule] pretreatment could reduce the mortality rate in CLP-induced sepsis. After CLP operation, i.e., sham group (*n* = 6), CLP-control group (*n* = 20), and CLP-probiotics group (*n* = 14), the mortality of mice was monitored every day until the seventh day.

### Collection of Serum, Peritoneal Lavage Fluid, and Ileal Tissues

After 72 h of CLP surgery, the mice were sacrificed and serum was firstly collected from the eyeballs of all mice to measure the serum cytokine levels. Then, pre-cooled PBS was used to repeatedly wash the abdominal cavity and the peritoneal lavage fluid was collected. Next, the abdominal cavity was opened using a midline incision and the ileal tissues were washed twice with ice cold PBS to reduce blood contamination.

### Histological Analysis of Ileal Tissues

The paraformaldehyde-fixed and paraffin-embedded tissues were cut into 5 μm-thick sections, followed by deparaffinizing in xylene and dehydrating with graded ethanol. After washing with PBS twice, HE staining was performed histological scoring was done by two trained investigators in a double blind study. A histopathological score was calculated according to the scoring criteria as Chiu’s previous description (Gekara and Weiss, 2007): Grade 0, normal intestinal mucosa villi; Grade 1, increase of spaces against intestinal mucosal epithelium, with capillary congestion and villus swelling; Grade 2, expansion of spaces under intestinal mucosal epithelium, with minor separation between epithelial layer and tunicae propria; Grade 3, extensive expansion of spaces in intestinal mucosal epithelium, with broken villus tips; Grade 4, villus breakage and shedding of lamina propria, and exposure of lamina propria capillaries; Grade 5, incomplete lamina propria, with bleeding and ulcers.

### Cytokine Assay

The ileal tissues were homogenized with the PBS, after sonication and centrifugation at 5,000 rpm for 10 min, the supernatant was utilized to measure the levels of TNF-α, IL-6, and IL-10, also the serum levels were measured using the TNF-α, IL-6, and IL-10 test kit (Beijing Roche Pharmaceutical Co., Ltd., China) according to the manufacturer’s instructions.

### Western Blotting (WB) Analysis

Radioimmunoprecipitation assay (RIPA) lysis solution (Beyotime, China), containing 50 mM Tris-HCl (pH 7.5), 150 mM NaCl, 0.1% Nonidet P-40, and a mixture of protease inhibitors, was applied to extract the total protein of three independent samples from different treatment groups for 20 min on ice. After centrifugation at 12,000 rpm for 10 min at 4°C, the insoluble pellets were removed, and the total protein concentration in the supernatant was measured using a BCA protein assay kit (Thermo Scientific, United States). Samples containing equal amounts of protein (30 μg) per lane were fractionated based on their molecular weight by 8–10% sodium dodecyl sulfate-polyacrylamide gels for electrophoresis (SDS-PAGE) and transferred onto polyvinylidene difluoride (PVDF) membranes at a constant current of 200 mA for 60 min. After blocking with 5% non-fat milk in Tris-buffered saline with 0.1% Tween-20 (TBST) for 2 h at room temperature, the membranes were incubated with primary antibodies against β-actin (1:5000, as a reference control; Abmart, United States), TGF-β1 (1:1000; Abcam, United States), CD11c (1:500; Proteintech, United States), CD206 (1:500; Proteintech, United States), p-AKT (1:1500; Abcam, United States), Akt (1:2000; Abcam, United States), Claudin (1:500; Sigma, Shanghai), Occludin (1:1000, Sigma, Shanghai), and ZO-1 (1:500, Sigma, Shanghai) overnight at 4°C. Subsequently, the membranes were extensively washed with TBST and incubated with the corresponding secondary antibodies (including goat anti-mouse IgG and goat anti-rabbit IgG, 1:2000) for 1 h at room temperature, and finally visualized by enhanced chemiluminescence reagents (Beyotime, China). The relative expression level of proteins were analyzed using ImageJ software.

### Analysis of Immunohistochemistry (IHC)

Paraformaldehyde-fixed and paraffin-embedded ileal specimens were used for analysis of IHC for CD68 detection. Briefly, the 5-μm sections were deparaffinized in xylene and dehydrated with graded ethanol. After washing with PBS twice, endogenous peroxidase activity was eliminated with 3% hydrogen peroxide (H_2_O_2_) in methanol for 15 min at room temperature. Next, the slides were heated at 100°C in a microwave oven for 20 min with citric acid buffer for antigen retrieval and then cooled for 20 min at room temperature. The slides were blocked with 2% bull serum albumin (BSA) at 37°C for 1 h and incubated with the primary antibodies diluted in blocking solution (Anti-CD68, 1:150 dilution; Abcam, United States) overnight at 4°C. The slides were washed with PBS three times and incubated with bio-tinylated goat anti-rabbit IgG at 37°C for 30 min. Subsequently, the slides were washed with PBS again and incubated with horseradish peroxidase-conjugated streptavidin for 5 min at room temperature, followed by immunodetection with diaminobenzidine (DAB). Finally, the slides were rinsed with PBS for 5 min, counterstained with Mayer’s hematoxylin for 1 min, dehydrated in a graded series of alcohol and sealed with cover slips. Images were visualized and captured under a light microscope.

### Toluidine Blue Staining

The ileal tissue slices were baked at 65°C for 20 min and dewaxed in xylene. Then, the slices were immersed in toluidine blue solution (Sigma, United States) for 30 min staining. After dehydration in graded ethanol, the slices were sealed and the MCs were examined under light microscope.

### Histamine Assay

The abdominal cavity lavage was centrifuged and the supernatant was applied to quantify histamine concentration with histamine ELISA kits (Elabscience Biotech, United States), according to the manufacturer’s instructions. The histamine concentration in serum was determined by a same kit. All experiments were performed in triplicates.

### Statistical Analysis

Statistical analysis was performed using SPSS 18.0 software (IBM SPSS, United States) and GraphPad Prism 5.0 software (GraphPad Software, Inc., United States). All data is presented as mean ± the standard error of the mean (SEM) or median with interquartile range depending on the distribution and type of the data. Difference was examined using an unpaired, two-tailed Student’s *T*-test or assessed using either analysis of variance (ANOVA) followed by Bonferroni method or the Kruskal–Wallis test followed by Dunn’s multiple comparison method. The value of *P* < 0.05 was considered as statistical significance.

## Results

### Pretreatment of LCBE Enteric-Coated Capsules Reduced Mortality in Septic Mice

**FIGURE 1:**
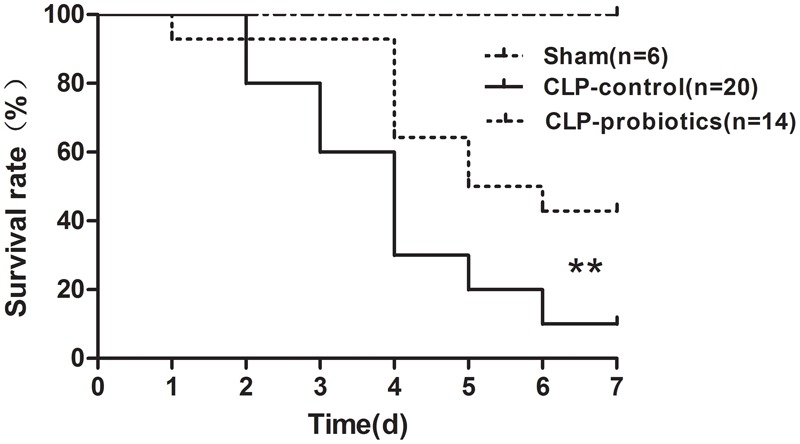
Survival of septic C57BL/6J mice with or without LCBE treatment. Seven-week-old C57BL/6J mice pretreated with LCBE (*n* = 14) or normal saline (*n* = 26) for 1 week. All of C57BL/6J mice treated with LCBE and 20 of C57BL/6J mice treated with normal saline were subjected to CLP surgery, while the rest six of C57BL/6J mice treated with normal saline were carried out a sham laparotomy. After surgery, all mice were monitored for 7 days and survival of groups were compared. ^∗∗^*P* < 0.01 (CLP-probiotic vs. CLP-control).

To determine whether LCBE enteric-coated capsules pretreatment could decrease mortality rate in CLP-induced septic mice, 14 mice with LCBE enteric-coated capsules pretreatment and 20 control mice were subjected to CLP surgery, and that six mice were subjected to sham surgery. All mice were monitored for 7 days after surgery. The results showed that six sham group mice survived (Figure 1), and the survival in septic mice pretreated with LCBE enteric-coated capsules was significantly higher than that in control septic mice (*P* < 0.01), suggesting that probiotics could improve the survival of CLP-induced septic mice.

### LCBE Enteric-Coated Capsules Reduced Ileal Injury in Septic Mice

**FIGURE 2:**
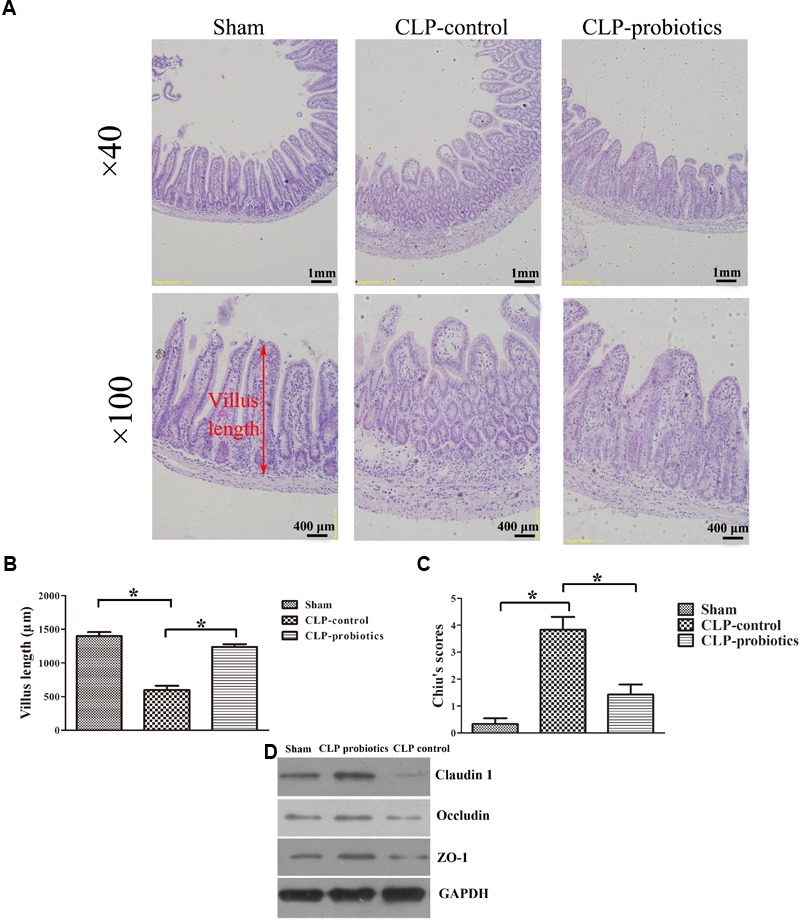
Histological analysis of intestines and injury scores in septic mice with or without LCBE treatment. **(A)** Representative images of HE staining sections of ileal in sham, CLP-control and CLP-probiotic groups under 40× and 100× magnification. **(B)** Villus length was determined to evaluate the impairments of ileal quantified in 12 oriented crypt-villus units. LCBE enteric-coated capsule pretreatment reduced the villus length. **(C)** Intestinal injury scores were evaluated using the Chiu’s grading system. **(D)** The expression of TJ proteins (ZO-1, claudin-1 or occludin) in ileal tissues were analyzed by Western blotting. All data were expressed as means ± SEM. *N* = 6 mice/group; ^∗^*P* < 0.05.

To assess the effects of LCBE enteric-coated capsules on ileal mucosal injury and integrity in CLP-induced sepsis, we measured villus length and calculated Chiu’s scores. As shown in Figure 2, increased histopathological changes were observed in CLP-treated mice, but not in sham mice, and pretreatment with LCBE enteric-coated capsules ameliorated the intestinal injury. Additionally, CLP surgery caused a significant decrease in villus length (205.5 ± 26.64 mm) and an apparent increase in Chiu’s score (3.833 ± 0.4773) as compared to sham group (villus length was 402 ± 40.39 mm and Chiu’s score was 0.3333 ± 0.2108) (*P* < 0.05). However, the villus length (302.7 ± 17.45 mm) was markedly elevated in probiotic-pretreated group (*P* < 0.05) compared to CLP-control group. The Chiu’s score (1.429 ± 0.3689) was significantly reduced in probiotic-pretreated group compared to CLP-control group. TJ proteins are important molecules for determination of the intestinal epithelial barrier, next, we measured the TJ proteins expression in ileal tissues. As shown in Figure 2D, TJ proteins (ZO-1, claudin-1 and occludin) expression was decreased in CLP-induced septic mice, but could be recovered by probiotic treatment. These results indicated that probiotics could ameliorate the histopathological injuries of ileal tissues in CLP septic mice.

### LCBE Enteric-Coated Capsules Suppressed the Expression of Pro-inflammatory Cytokines IL-6 and TNF-α, but Not for Anti-inflammatory TGF-β1 and IL-10

**FIGURE 3:**
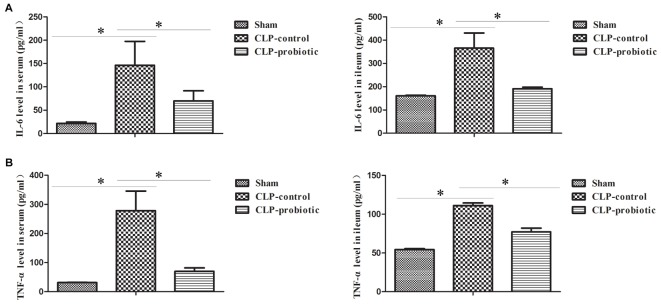
The levels of pro-inflammatory IL-6 and TNF-α in the septic mice with or without LCBE treatment. **(A)** IL-6 level in serum and ileal tissues were examined by ELISA. LCBE enteric-coated capsules decreased the expression of IL-6. **(B)** TNF-α level in serum and ileal tissues were examined by ELISA. LCBE enteric-coated capsule treatment decreased the expression of TNF-α. All data were presented as means ± SEM. *N* = 6 mice/group; ^∗^*P* < 0.05.

**FIGURE 4:**
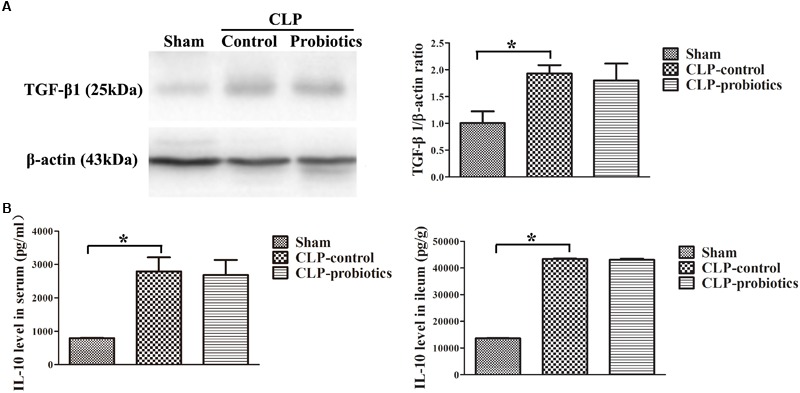
The levels of anti-inflammatory TGF-β1 and IL-10 in the septic mice with or without LCBE treatment. **(A)** The protein expressions of TGF-β1 in each group were determined by Western blotting assay (left panel). The quantitative analysis of TGF-β1 bands was presented in right panel. **(B)** IL-10 levels in serum and ileal tissues were detected by ELISA. All data were showed as means ± SEM. *N* = 6 mice/group; ^∗^*P* < 0.05.

As illustrated in Figure 3, the levels of IL-6 and TNF-α in serum and intestinal samples of CLP mice were remarkably higher than those in sham group (*P* < 0.05). Moreover, when pre-treating with LCBE enteric-coated capsules, the expressions of IL-6 and TNF-α in serum and ileal tissues of CLP mice were significantly decreased. Additionally, the protein level of TGF-β1 was examined in intestinal samples by Western blot. The result indicated that TGF-β1 expression was significantly up-regulated in CLP-control group as compared to sham group (*P* < 0.05) but there were no significant difference between CLP-control group and CLP-probiotic group (Figure 4A). Similarly, IL-10 level were measured in serum and intestinal, the data indicated that the expression of IL-10 in CLP group was higher than that in sham group, but no significant difference was observed between CLP-control group and CLP-probiotic group (Figure 4B). These results implied that probiotics have a protective role by decreasing inflammatory component in CLP-induced sepsis model.

### LCBE Enteric-Coated Capsules Regulated Macrophage Activation and Transformation

**FIGURE 5:**
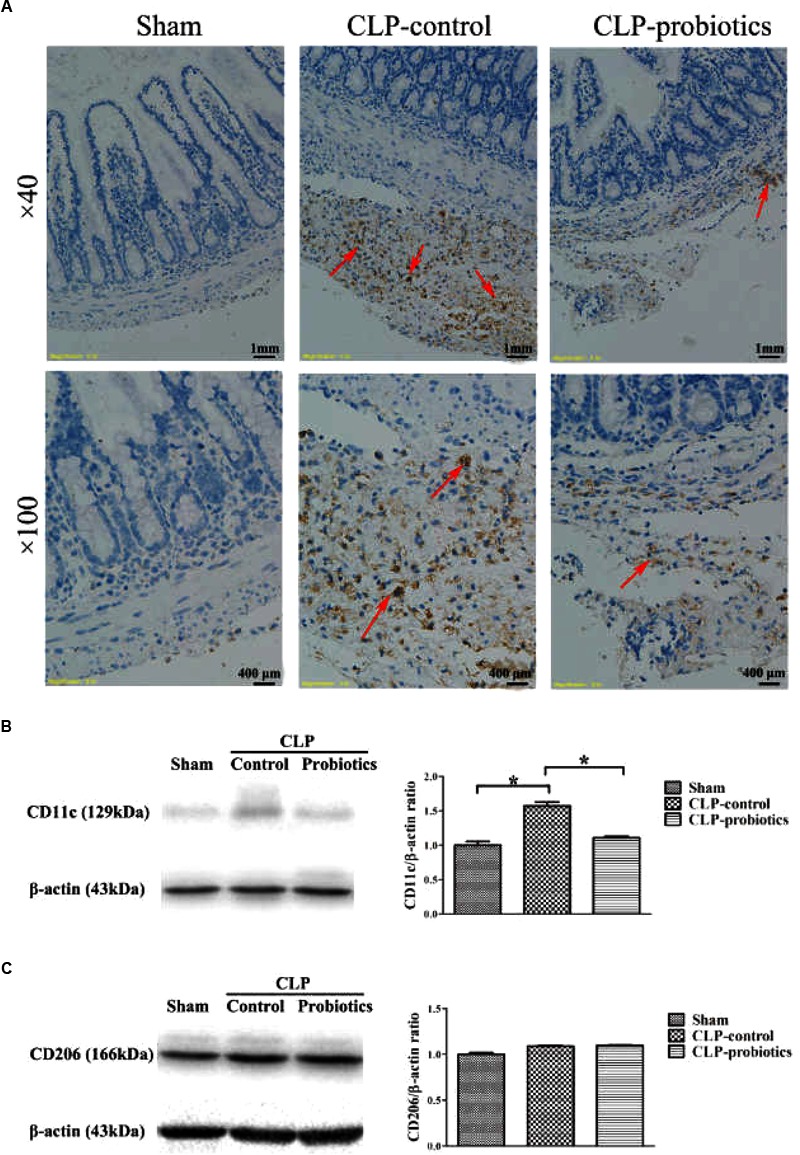
The effect of LCBE pretreatment on the macrophage activation and transformation. **(A)** CD68was stained in ileal tissues by IHC assay under 40× and 100× magnifications. **(B)** The protein expression of CD11cwas examined by Western blotting. Representative photographs of CD11c and β-actin blots were displayed in left panel. The bar graph was presented in right panel. **(C)** The protein expression of M2 type macrophage marker CD206 was examined. Representative photographs of CD206 and β-actin blots were exhibited in left panel. The bar graph was presented in right panel. All data were showed as means ± SEM. *N* = 6 mice/group; *N* = 6 mice/group; ^∗^*P* < 0.05.

Immunohistochemistry staining analysis showed a positive staining of CD68 molecule in CLP-control group, while the staining of CD68 molecule was apparently weak in CLP-probiotic group (Figure 5A), indicating that LCBE enteric-coated capsules inhibited macrophages activation. Furthermore, we examined the expression of CD11c, a marker of M1 type cell (Jingjing et al., 2013), and found that CD11c expression was significantly increased in CLP mice compared to sham group (*P* < 0.05, Figure 5B), But it was markedly decreased in CLP-probiotic group compared to CLP-control group (*P* < 0.05). In addition, no significant change in CD206, a marker of M2 type cell (Wu et al., 2016; Dou and Bennett, 2017), was observed among sham, CLP-control and CLP-probiotic groups (Figure 5C). Therefore, these findings suggest that probiotics exert their protective effects through suppression of macrophage activation and transformation.

### LCBE Enteric-Coated Capsules Inhibited MC Degranulation and Histamine Secretion

**FIGURE 6:**
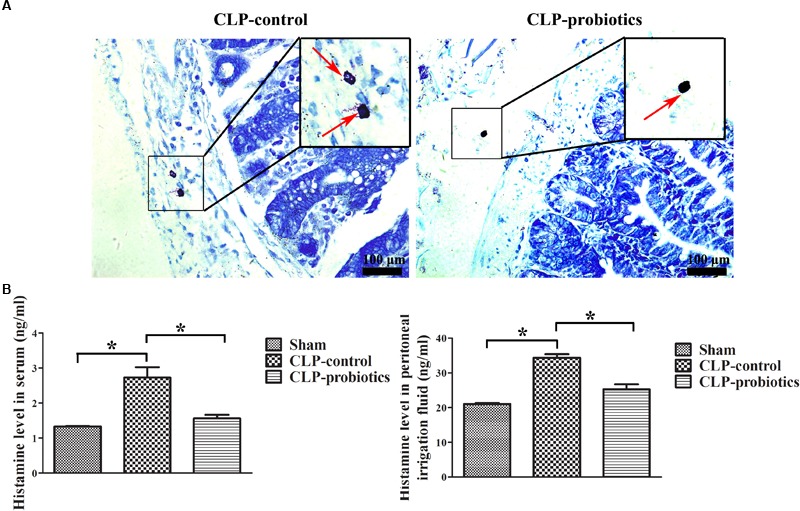
The effect of LCBE pretreatment on MCs degranulation and histamine secretion. **(A)** The representative images of toluidine blue staining to identify the degranulation of MCs. The degranulation of MCs was marked by red arrow. **(B)** The histamine contents in serum and peritoneal lavage fluid were measured by ELISA. All data were illustrated as means ± SEM. *N* = 6 mice/group; ^∗^*P* < 0.05.

Toluidine blue staining showed that the granulation of MCs in CLP-control group was obvious, but not in CLP-probiotic group (Figure 6A). The levels of histamine in serum and peritoneal lavage fluid in CLP-control group were significantly higher than those in sham group (*P* < 0.05). Nevertheless, the levels of histamine in serum and peritoneal lavage fluid in CLP-probiotic group were lower than those in CLP-control group (*P* < 0.05, Figure 6B). Thus, these results suggest that probiotics exert their protective effect through inhibition of the MCs degranulation.

### LCBE Enteric-Coated Capsules Promoted the AKT Activation

**FIGURE 7:**
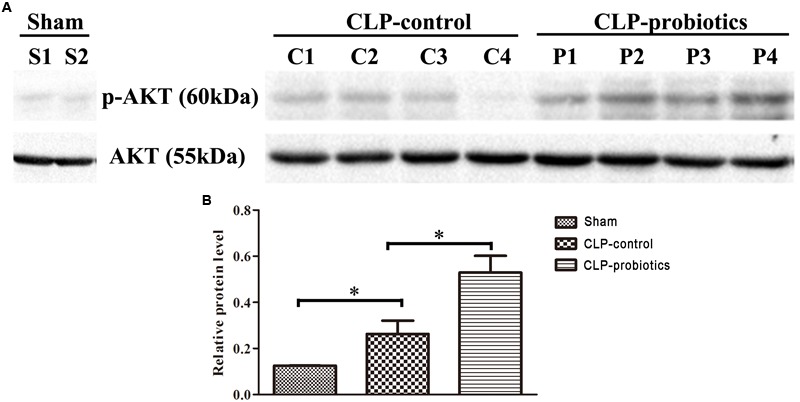
The effect of LCBE pretreatment on p-AKT and total AKT expression in the ileal tissues of the septic mice. **(A)** Total AKT and phosphorylated AKT (p-AKT) in the ileal tissues of three groups were determined by Western blotting. **(B)** Relative levels (p-AKT/AKT) of each group were quantitatively analyzed. All data were illustrated as means ± SEM. *N* = 4 mice/group; ^∗^*P* < 0.05.

In order to investigate the molecular mechanisms mediated by LCBE enteric-coated capsules in the gastrointestinal system of septic mice, the activation of AKT and phosphorylated-AKT (p-AKT) in intestinal samples were determined by Western blotting analysis. The results showed that p-AKT level in CLP-probiotic group were elevated when compared with CLP-control group (*P* < 0.05, Figure 7), indicating that AKT signaling pathway was regulated by probiotic pretreatment.

## Discussion

Sepsis is a common and life-threatening systemic illness with high mortality in critically ill patients (Vincent et al., 2011). Despite significant efforts have been made in diagnostic and therapeutic approaches of sepsis in recent years, there has been no effective drugs (Richter et al., 2017). Therefore, understanding the underlying mechanisms of sepsis could reduce the mortality and morbidity associated with this disease. Previous studies have demonstrated that impaired function of vital organs, especially in gastrointestinal tract, is one of the major causes of septic death (Kell and Pretorius, 2017). For example, Jingjing et al. (2013) confirmed that there were a positive correlation between the severity of gastrointestinal dysfunction and prognosis of sepsis. Additionally, there is an imbalance between proinflammatory and anti-inflammatory response imbalance resulting in tissue hypoperfusion and ischemia, and impaired intestinal mucosal barrier function and subsequent death. Due to disruption of intestinal barrier, the intestinal wall becomes leaky and permeable to various macromolecular substances, bacterial products and microbes penetrated through the intestinal wall into the adjacent tissue or blood, and exuberating sepsis (Wu et al., 2016). Hence, it is important to maintain intestinal barrier function for the prevention of sepsis occurrence and progression. Over the past decades, it has been reported that probiotics, living commensal microorganisms colonized in intestinal tract benefit to the host, so the clinical therapeutic effects of probiotics are gradually received a special attention in gastrointestinal system’s diseases (Dylag et al., 2014; Sanchez et al., 2017).

A great deal of evidence has reported that probiotics exerted its key role in regulating the body’s immune system, inhibiting intestinal infections and maintaining gastrointestinal microbial flora balance (Sarowska et al., 2013; Dylag et al., 2014). Moreover, it could alleviate the incidence of MODS and promote the recovery of various organs of the body during MODS (Kell and Pretorius, 2017), but its potential mechanisms of probiotic in sepsis progression remains unknown. In this study, our data presented that LCBE enteric-coated capsules could not only reduce the mortality of CLP-induced sepsis, but also decrease ileal damages. Furthermore, LCBE enteric-coated capsules could down-regulate pro-inflammatory IL-6 and TNF-α expression in serum and intestinal tissues, but not for anti-inflammatory TGF-β and IL-10 expression. That is to say, LCBE enteric-coated capsules could reduce inflammatory response through decreasing the expression of pro-inflammatory factors and not altering the expression of anti-inflammatory factors.

Macrophages, an important type of immune cells, can be polarized into type M1 macrophages and type M2 macrophages, which have an opposite function in normal physical situation (Martinez, 2011). M1 macrophages show a strong ability of pro-inflammation and antigen presentation and play a host immune clearance function for pathogens and tumor cells, while M2 macrophages emerge a resistant to inflammation, promote wound healing and fibrosis, repair tissues and accelerate tumor growth and infiltration (Xaus et al., 2001; Kozicky and Sly, 2015). Therefore, the polarization type of macrophages might influence different immune condition of body. It has been reported that macrophages could regulate innate immunity during sepsis (Kozicky and Sly, 2015), we thereby tested the changes of macrophages in this study. Our results revealed that LCBE enteric-coated capsules could inhibit macrophage activation and prevent them from macrophages to M1 type cell polarization. M1 macrophages usually released pro-inflammatory, such as IFN-γ, TNF-α, IL-1β, and IL-6 cytokines, to against the microbial inflammation or tissue inflammation damages (Martinez, 2011). LCBE enteric-coated capsules could suppress polymicrobial sepsis with the characteristics of macrophage activation and transformation (M1 polarization).

Mast cells are widely distributed in the connective tissue around the mucosal capillaries of the skin, respiratory tract, digestive tract and others (Galli et al., 1999, 2005). MCs could trigger the innate immune response through production of chemoattractants for neutrophils, which are crucial to bacterial clearance in the peritoneal cavity after CLP (Echtenacher et al., 1996; Mallen–St Clair et al., 2004). Gekara and Weiss (2007) have demonstrated that the infectious degree significantly aggravated in MCs-deficient mice, which suggest that MCs might strongly resist pathogen infection. Furthermore, Echtenacher et al. (1996) also observed that the mortality rate in MCs-lacked W/Wv mice was significantly higher than that in normal mice, while the mortality rate was decreased when intraperitoneally transplanting normal MCs into W/Wv-lacked mice. Therefore, MCs plays an important role during the development of various diseases. Our data in this study indicated that LCBE enteric-coated capsules could inhibit the degranulation of MCs, thereby suggesting the role of probiotics in regulation of MCs. Also, histamine levels released from MCs were further examined. Histamine levels were not only recognized as a “gold standard” for clinical diagnosis in acute allergic diseases, but also used as a marker of MCs degranulation (Busse and Smith, 2017; Yamazaki et al., 2017). The data suggests that levels of histamine, which is identified as an aggravating mediator that accelerate the development of major end-organ (that is, lung, liver, and kidney) injury, in both serum and peritoneal lavage fluid were decreased after using of LCBE enteric-coated capsules in CLP-induced sepsis. Consistently, we found that LCBE enteric-coated capsules could inhibit the degranulation of MCs in CLP-induced sepsis.

To elucidate the molecular mechanisms of LCBE-mediated protective effects, we studied the signaling pathway promoting the polarization of macrophages. Previous studies indicated that the regulated signaling pathways in polarization of macrophages mainly included JAK/STAT signaling pathway, PI3K/Akt signaling pathway, C-Jun N-terminal kinase (JNK) signaling pathway and Notch signaling pathway. Among these signaling pathways, we studied the changes of PI3K/Akt signaling pathway activation that regulate the activation of M1 or M2 macrophages and a variety of cellular behavior, such as cell activity, proliferation, differentiation and metabolism (Xaus et al., 2001). We found that LCBE enteric-coated capsules increased the AKT phosphorylation in CLP sepsis, suggesting that the protective role of probiotics might be implemented by up-regulation of AKT signaling pathway. Moreover, TJ proteins, including claudin-1, occluding and ZO-1, are critical regulation factors of barrier functions and cytoskeletal mediated leak pathway (Shen et al., 2011). Studies have proved that in some intestinal inflammatory diseases, the intestinal mucosal barrier was disabled by loss of expression and intercellular junctions of TJ proteins (Ivanov et al., 2004; Clark et al., 2006). Therefore, the protective of intestinal mucosal barrier may serve an important role in improving survival during sepsis. In the present study, we found that the LCBE enteric-coated capsules treatment can inhibit the loss of TJ proteins in CLP-induced-sepsis mice. Thus, probiotics can promote survival in sepsis through maintain the integrality of intestinal barrier functions.

Collectively, our study is the first to demonstrate that probiotic pretreatment improves survival and reduces intestinal injury and inflammation in CLP-induced sepsis. Further, Probiotics exhibit their anti-inflammatory effect by decreasing macrophages to M1 type polarization and inhibiting MCs degranulation. Moreover, LCBE enteric-coated capsules exert their protective role is through the inhibition of AKT signaling. Thus, based on our results we propose that probiotics may could be potential agents for the treatments of sepsis in critically ill patients.

## Author Contributions

LG, MM, and YW designed the study and wrote the manuscript. FL, YJ, and XC performed the experiments and collected the data. GW, CW, and XG analyzed the data and helped in editing the article.

## Conflict of Interest Statement

The authors declare that the research was conducted in the absence of any commercial or financial relationships that could be construed as a potential conflict of interest. The reviewer, QM, declared a shared affiliation, with no collaboration, with one of the authors, GW, to the handling Editor at the time of review.
